# Clinical adverse events to dexmedetomidine: a real-world drug safety study based on the FAERS database

**DOI:** 10.3389/fphar.2024.1365706

**Published:** 2024-07-02

**Authors:** Feng Liu, Jing-xuan Zheng, Xiao-dan Wu

**Affiliations:** Department of Anesthesiology, Shengli Clinical Medical College, Fujian Provincial Hospital, Fujian Medical University, Fuzhou, China

**Keywords:** dexmedetomidine, FDA adverse event reporting system, real-world data analysis, adverse events, adverse drug reaction

## Abstract

**Objective:**

Adverse events associated with dexmedetomidine were analyzed using data from the FDA’s FAERS database, spanning from 2004 to the third quarter of 2023. This analysis serves as a foundation for monitoring dexmedetomidine’s safety in clinical applications.

**Methods:**

Data on adverse events associated with dexmedetomidine were standardized and analyzed to identify clinical adverse events closely linked to its use. This analysis employed various signal quantification analysis algorithms, including Reporting Odds Ratio (ROR), Proportional Reporting Ratio (PRR), Bayesian Confidence Propagation Neural Network (BCPNN), and Multi-Item Gamma Poisson Shrinker (MGPS).

**Results:**

In the FAERS database, dexmedetomidine was identified as the primary suspect in 1,910 adverse events. Our analysis encompassed 26 organ system levels, from which we selected 346 relevant Preferred Terms (PTs) for further examination. Notably, adverse drug reactions such as diabetes insipidus, abnormal transcranial electrical motor evoked potential monitoring, acute motor axonal neuropathy, and trigeminal cardiac reflex were identified. These reactions are not explicitly mentioned in the drug’s specification, indicating the emergence of new signals for adverse drug reactions.

**Conclusion:**

Data mining in the FAERS database has elucidated the characteristics of dexmedetomidine-related adverse drug reactions. This analysis enhances our understanding of dexmedetomidine’s drug safety, aids in the clinical management of pharmacovigilance studies, and offers valuable insights for refining drug-use protocols.

## 1 Introduction

Dexmedetomidine, a potent α2 adrenergic receptor agonist with high selectivity, facilitates perioperative sedation, anxiolytic and analgesic effects by targeting postsynaptic α2 receptors (Carollo et al., 2008). Although it was initially approved only for short-term (less than 24 h) sedation in adult intensive care units (Venn et al., 1999), its use in clinical practice has ranged from sedation of non-intubated patients to adjunctive use in surgical anesthesia over the past few years (Paris and Tonner, 2005; Liu et al., 2021; Kong et al., 2023). Dexmedetomidine induces a unique mode of sedation that mimics natural sleep and therefore facilitates perioperative sedation by minimal respiratory depression (Akeju et al., 2018). Recent clinical trials have highlighted its efficacy in managing acute agitation in patients with schizophrenia and bipolar disorder (Citrome et al., 2022; Preskorn et al., 2022). Meanwhile, dexmedetomidine’s molecular mechanisms of organ protection through its anti-inflammatory and activation of specific anti-apoptotic signaling pathways are likewise the focus of current clinical researchers (Bao and Tang, 2020). However, while the clinical use of dexmedetomidine is growing, its associated adverse effects, including bradycardia, delayed recovery, respiratory and circulatory depression require significant attention (De Cassai et al., 2021; Baumgartner et al., 2023). Furthermore, despite existing clinical trials and basic research providing insights into dexmedetomidine’s safety profile, a more comprehensive analysis of its adverse effects in real-world clinical settings remains necessary.

Data mining techniques, including signal detection algorithms, are increasingly utilized to scrutinize medical databases, analyzing extensive data to uncover potential drug-adverse event associations that might not be evident in clinical trials (Wilson et al., 2004; Chakraborty, 2015). The FDA’s Adverse Event Reporting System (FAERS) is among the largest databases for post-market safety monitoring of approved drugs and biologics (Xu and Wang, 2014). This public database platform encourages multiple parties, including healthcare professionals, consumers, and pharmaceutical companies, to assess the real-world safety of drugs post-market through spontaneous reporting of adverse drug events.

This study aims to analyze dexmedetomidine associated adverse drug reaction signals using various disproportionate analysis methods, including the Reporting Odds Ratio (ROR) (Rothman et al., 2004), Proportional Reporting Ratio (PRR) (Evans et al., 2001), Bayesian Confidence Propagation Neural Network (BCPNN) (Bate et al., 1998), and Multi-Item Gamma Poisson Shrinker (MGPS) algorithms (Almenoff et al., 2006). Employing multiple disproportionality analysis methods in retrospective pharmacovigilance studies enhances the confidence in results and rigorously screens for significant positive signals. The objective is to provide valuable data on the safety of dexmedetomidine administration to support more prudent use in the future, offering a reliable evidence-based foundation for expanding its clinical applications.

## 2 Materials and methods

### 2.1 Study design and data source

This observational, retrospective study conducted a disproportionality analysis, which using data from the publicly available FAERS database, spanning from the first quarter of 2004 to the third quarter of 2023. The data, comprising adverse drug reaction events, were extracted from 79 quarterly ASCII data packages and analyzed using R software (version 4.2.2) after thorough data cleaning.

### 2.2 Data extraction and descriptive analysis

The FAERS database comprises seven data files: patient demographics (DEMO), drug information (DRUG), adverse event information (REAC), patient outcome information (OUTC), report source information (RPSR), medication therapy date information (THER), and medication indications (INDI). Adverse drug reactions in FAERS are categorized and standardized according to the Medical Dictionary for Regulatory Activities (MedDRA) (Brown, 2004). In FAERS, each report employs MedDRA’s preferred terms (PTs), which are linked to various levels such as High-Level Terminology (HLT), High-Level Group Terminology (HLGT), and System Organ Class (SOC). This study adheres to MedDRA’s definitions.

In this study, records related to dexmedetomidine were identified using “dexmedetomidine” and its trade name “Precedex” as keywords, with “role_cod” set to PS (Primary Suspect). To eliminate duplicate reports, as recommended by the FDA, we sorted the DEMO table’s PRIMARYID, CASEID, and FDA_DT fields by CASEID and FDA_DT. We retained the report with the latest FDA_DT for each CASEID, and in cases of identical CASEID and FDA_DT, the report with the largest PRIMARYID was kept.

**FIGURE 1:**
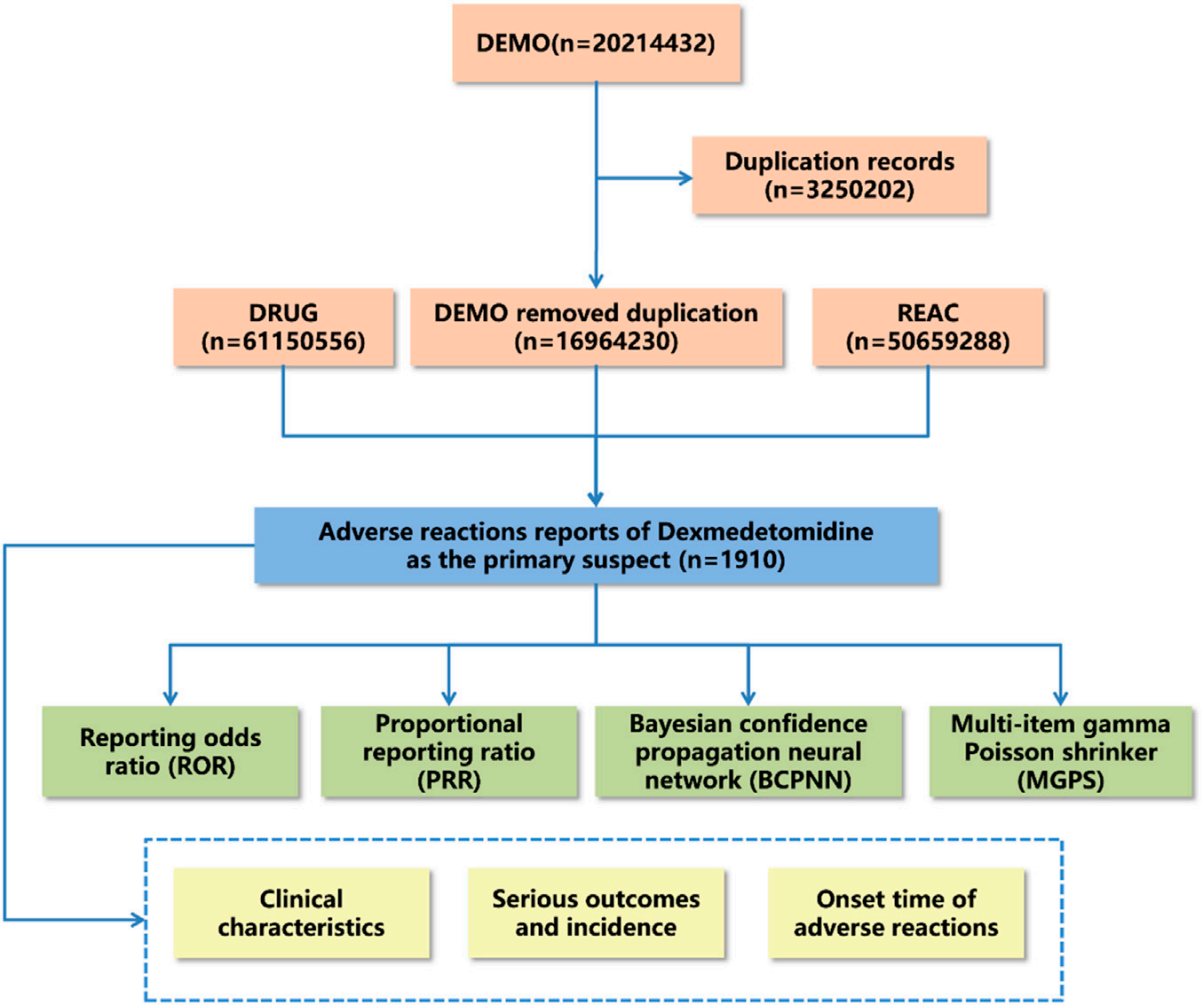
Flow diagram for the selection of adverse events associated with dexmedetomidine from FAERS database.

Adverse drug reaction reports were statistically analyzed to describe clinical characteristics such as gender, age, reporter type, reporting region, report timing, and outcomes. Notably, serious outcomes encompassed death, life-threatening conditions, hospitalization, disability, and other significant health impacts. However, the count of serious outcomes may surpass the total report count, as some reports indicated multiple serious outcomes. The methodology, including data extraction, processing, and analysis, is illustrated in Figure 1.

### 2.3 Statistical analysis

Given that the Faers database consists of spontaneous reports and lacks complete real-world adverse drug reaction denominator data, directly calculating the incidence of adverse drug reaction events is not feasible. However, disproportionality analysis effectively identifies signals of adverse drug reaction events in retrospective pharmacovigilance studies (Almenoff et al., 2007). To overcome the limitations of single algorithms and enhance the reliability and accuracy of data mining results, we employed multiple algorithms for data analysis. Thus, we used disproportionality methods, including Reporting Odds Ratio (ROR), Proportional Reporting Ratio (PRR), Bayesian Confidence Propagation Neural Network (BCPNN), and Multi-Item Gamma Poisson Shrinker (MGPS), to detect adverse drug event signals in the present study.

ROR and PRR methods are designed to identify the excessive frequency of adverse event reports, indicating potential risks associated with dexmedetomidine (Evans et al., 2001; Rothman et al., 2004). BCPNN is a valuable adjunct for accurately detecting potential associations between drugs and adverse events (Bate et al., 1998). MGPS offers a comprehensive analysis by quantifying adverse event signals, considering report counts and background risk (Almenoff et al., 2006). For high-frequency adverse event reporting, ROR is more applicable due to its ability to minimize bias and assess relative risk through the rational selection of control samples (Rothman et al., 2004). Additionally, MGPS is better suited for detecting rare adverse drug reactions because it is less confounded by demographic factors, and provides high specificity and more stable results with fewer reports (Almenoff et al., 2006). The methodologies, including detailed formulas and procedures, are outlined in Supplementary Tables S1, S2. For initial screening, preferred terms (PTs) with report counts ≥3 were selected, utilizing MedDRA (Medical Dictionary for Regulatory Activities) PT and System Organ Class (SOC) for coding, classifying, and localizing the signals to analyze the specific SOC involved in the adverse event signals.

In summary, disproportionately positive signals were defined according to the following criteria: the number of reported cases was three or more, the lower limit of the 95% confidence interval between ROR and PRR was greater than one, the chi-square value (*χ* ^2^) was at least four, IC025 was greater than zero, and EBGM was greater than two (Kinoshita et al., 2020).

In order to enhance the reliability of the findings, separate disproportionate analyses were conducted, stratified by patient age (<18 years, 18–65 years, >65 years), gender (male, female), and weight (<50 kg, 50–100 kg, >100 kg).

## 3 Results

### 3.1 Basic information of dexmedetomidine related adverse events reports

As of the third quarter of 2023, 1,910 adverse events reports related to dexmedetomidine were analyzed by applying specific selection criteria. The data processing flow is depicted in Figure 1. The analysis revealed an increasing trend in dexmedetomidine-associated adverse events cases annually from 2004 to 2023 Q3, with 223 cases reported in 2023 alone, the highest yearly count, representing 11.68% of the total. Notably, adverse events reports from the last 5 years comprised 53.72% of the total. Female patients were more frequently reported than male patients (48.8% vs. 28.7%) in dexmedetomidine-related adverse events. The majority of cases were in the 18–64 age group, accounting for 31.2%. Medical practitioners, predominantly physicians, submitted most reports, totaling 589 (30.8%). The United States was the primary reporting country, contributing 43% of reports. Regarding serious outcomes, events leading to or prolonging hospitalization were most common (495 cases, 19.9%), followed by life-threatening events (342 cases, 13.8%). Most dexmedetomidine adverse drug reactions occurred within 7 days of dosing. These findings (detailed in Table 1) offer insights into the demographic and clinical characteristics of dexmedetomidine-related adverse events reports, aiding in the evaluation and optimization of clinical dosing regimens.

**TABLE 1:** Basic information on adverse reactions related to dexmedetomidine from the FAERS database (2004 to 2023Q3).

Characteristics	Number of events (%)
Gender	
Female	549 (28.7%)
Male	933 (48.8%)
Unknown	428 (22.4%)
Age	
≤17	300 (15.7%)
18∼64	596 (31.2%)
65∼85	367 (19.2%)
≥86	19 (1.0%)
Missing	628 (32.9%)
Reporter	
Consumer	43 (2.3%)
Health professional	484 (25.3%)
Physician	589 (30.8%)
Other health-professional	463 (24.2%)
Pharmacist	275 (14.4%)
Registered nurse	1 (0.1%)
Missing	55 (2.9%)
Reported countries	
United States	821 (43.0%)
Australia	30 (1.57%)
Japan	436 (22.83%)
Other	623 (32.62%)
Reported year	
2004	12 (0.63%)
2005	11 (0.58%)
2006	33 (1.73%)
2007	20 (1.05%)
2008	30 (1.57%)
2009	38 (1.99%)
2010	23 (1.2%)
2011	13 (0.68%)
2012	23 (1.2%)
2013	59 (3.09%)
2014	62 (3.25%)
2015	67 (3.51%)
2016	178 (9.32%)
2017	171 (8.95%)
2018	144 (7.54%)
2019	209 (10.94%)
2020	180 (9.42%)
2021	206 (10.79%)
2022	208 (10.89%)
2023 Q1-Q3	223 (11.68%)
Serious outcomes	
Death	147 (5.9%)
Disability	24 (1.0%)
Hospitalization - initial or prolonged	495 (19.9%)
Life-threatening	342 (13.8%)
Adverse event occurrence time - medication date (days)	
0–7	137 (7.17%)
8–28	31 (1.62%)
29–60	5 (0.26%)
≥60	3 (0.16%)
Unknown	1734 (90.79%)

### 3.2 Signal mining for dexmedetomidine-related clinical adverse drug reactions

Adverse event signals associated with dexmedetomidine as the primary suspect were identified using ROR, PRR, BCPNN, and MGPS analyses. At the SOC level, dexmedetomidine was implicated in 26 categories, of which the top three most prevalent are cardiac organ disorders (*n* = 984; ROR 8.52; PRR 7.09; IC 2.82; EBGM 7.08), injury, poisoning and procedural complications (*n* = 766; ROR 1.59; PRR 1.50; IC 0.58; EBGM 1.50), and general disorders and administration site conditions (*n* = 641; ROR 0.67; PRR 0.71; IC −0.49; EBGM 0.71). Additionally, this study identified emerging adverse drug reactions not listed in the drug insert, including infections and infestations (*n* = 101; ROR 0.36; PRR 0.38; IC −1.41; EBGM 0.38), endocrine disorders (*n* = 79; ROR 6.20; PRR 6.12; IC 2.61; EBGM 6.12), and musculoskeletal and connective tissue disorders (*n* = 56; ROR 0.20; PRR 0.21; IC −2.28; EBGM 0.21). These findings (detailed in Table 2) underscore the importance of cautious dexmedetomidine administration in clinical practice, considering patient safety and pre-existing medical conditions.

**TABLE 2:** The adverse reactions of dexmedetomidine at the SOC level in FAERS database (2004 to 2023Q3).

System organ class	n	Percentage (%)	ROR (95% CI)	PRR (95% CI)	*χ* ^2^	IC (IC025)	EBGM (EBGM05)
Cardiac disorders	984	19.03	8.52 (7.95–9.13)	7.09 (6.70–7.50)	5,284.53	2.82 (1.16)	7.08 (6.68)
Injury, poisoning and procedural complications	776	15.00	1.59 (1.47–1.71)	1.50 (1.40–1.60)	143.21	0.58 (−1.08)	1.50 (1.41)
General disorders and administration site conditions	641	12.39	0.67 (0.62–0.73)	0.71 (0.66–0.76)	91.18	−0.49 (−2.16)	0.71 (0.66)
Nervous system disorders	464	8.97	1.05 (0.95–1.15)	1.05 (0.96–1.14)	0.98	0.06 (−1.60)	1.05 (0.96)
Investigations	462	8.93	1.48 (1.35–1.63)	1.44 (1.32–1.57)	66.21	0.53 (−1.14)	1.44 (1.33)
Respiratory, thoracic and mediastinal disorders	458	8.86	1.97 (1.79–2.16)	1.88 (1.72–2.05)	198.32	0.91 (−0.75)	1.88 (1.74)
Vascular disorders	288	5.57	2.67 (2.37–3.01)	2.58 (2.30–2.88)	283.92	1.37 (−0.30)	2.58 (2.33)
Psychiatric disorders	201	3.89	0.67 (0.58–0.77)	0.68 (0.60–0.78)	31.73	−0.55 (−2.22)	0.68 (0.61)
Gastrointestinal disorders	152	2.94	0.32 (0.28–0.38)	0.34 (0.29–0.40)	207.31	−1.54 (−3.20)	0.34 (0.30)
Infections and infestations	101	1.95	0.36 (0.30–0.44)	0.38 (0.31–0.46)	110.20	−1.41 (−3.08)	0.38 (0.32)
Renal and urinary disorders	97	1.88	0.97 (0.79–1.18)	0.97 (0.79–1.18)	0.12	−0.05 (−1.72)	0.97 (0.82)
Metabolism and nutrition disorders	92	1.78	0.81 (0.66–1.00)	0.82 (0.67–1.00)	3.92	−0.29 (−1.96)	0.82 (0.69)
Skin and subcutaneous tissue disorders	88	1.70	0.30 (0.25–0.38)	0.32 (0.26–0.39)	137.29	−1.66 (−3.33)	0.32 (0.27)
Endocrine disorders	79	1.53	6.20 (4.96–7.74)	6.12 (4.92–7.62)	338.90	2.61 (0.95)	6.12 (5.08)
Musculoskeletal and connective tissue disorders	56	1.08	0.20 (0.15–0.26)	0.21 (0.16–0.27)	180.18	−2.28 (−3.94)	0.21 (0.17)
Immune system disorders	52	1.01	0.92 (0.70–1.20)	0.92 (0.70–1.20)	0.40	−0.13 (−1.79)	0.92 (0.73)
Product issues	38	0.73	0.47 (0.34–0.65)	0.47 (0.34–0.65)	22.61	−1.08 (−2.75)	0.47 (0.36)
Pregnancy, puerperium and perinatal conditions	27	0.52	1.19 (0.81–1.74)	1.19 (0.82–1.73)	0.81	0.25 (−1.42)	1.19 (0.87)
Hepatobiliary disorders	26	0.50	0.55 (0.37–0.81)	0.55 (0.37–0.81)	9.67	−0.86 (−2.53)	0.55 (0.40)
Surgical and medical procedures	22	0.43	0.32 (0.21–0.49)	0.33 (0.21–0.49)	31.03	−1.62 (−3.28)	0.33 (0.23)
Eye disorders	21	0.41	0.20 (0.13–0.31)	0.21 (0.13–0.32)	65.71	−2.28 (−3.95)	0.21 (0.14)
Congenital, familial and genetic disorders	20	0.39	1.22 (0.79–1.89)	1.22 (0.79–1.89)	0.80	0.29 (−1.38)	1.22 (0.85)
Neoplasms benign, malignant and unspecified (incl cysts and polyps)	13	0.25	0.09 (0.05–0.16)	0.09 (0.05–0.16)	118.50	−3.43 (−5.10)	0.09 (0.06)
Blood and lymphatic system disorders	10	0.19	0.11 (0.06–0.21)	0.11 (0.06–0.21)	69.44	−3.12 (−4.79)	0.11 (0.07)
Ear and labyrinth disorders	2	0.04	0.09 (0.02–0.35)	0.09 (0.02–0.35)	18.80	−3.49 (−5.16)	0.09 (0.03)
Social circumstances	2	0.04	0.08 (0.02–0.33)	0.08 (0.02–0.33)	20.21	−3.58 (−5.25)	0.08 (0.03)

Our examination of Preferred Terms (PT) signals identified 346 significant PTs meeting the criteria of all four algorithms. These were ranked using the Empirical Bayesian Geometric Mean (EBGM) algorithm, with the top 30 PTs, each reported in three or more cases, presented in Table 3. Consistent with the drug specifications, the most common clinical adverse reactions were bradycardia, cardiac arrest and hypotension. The results indicated notable signal strength in conditions not listed in the drug instructions, such as transcranial electrical motor evoked potential abnormalities (*n* = 5; ROR 2723.16; PRR 2720.53; IC 11.06; EBGM 2129.33), acute motor axonal neuropathy (*n* = 10; ROR 1509.67; PRR 1506.76; IC 10.35; EBGM 1305.99) and trigeminal cardiac reflex (*n* = 7; ROR 1204.39; PRR 1202.76; IC 10.07; EBGM 1071.32). Additionally, the top five clinical adverse reactions with the highest case numbers following EBGM sequencing were diabetes insipidus (*n* = 75; ROR 389.00; PRR 383.37; IC 8.53; EBGM 368.97), arteriospasm coronary (*n* = 65; ROR 211.78; PRR 209.13; IC 7.68; EBGM 204.78), upper airway obstruction (*n* = 37; ROR 498.61; PRR 495.05; IC 8.88; EBGM 471.28), sinus arrest (*n* = 30; ROR 233.43; PRR 232.08; IC 7.82; EBGM 226.73), and sedation complications (*n* = 30; ROR 162.20; PRR 161.26; IC 7.31; EBGM 158.66).

**TABLE 3:** The top 30 clinical adverse reactions of dexmedetomidine ranked by EBGM at the PTs level in FAERS database (*n* ≥ 3, 2004 to 2023Q3).

Preferred terms	n	ROR (95% CI)	PRR (95% CI)	*χ* ^2^	IC (IC025)	EBGM (EBGM05)
Transcranial electrical motor evoked potential monitoring abnormal	5	2,723.16 (1,010.65–7,337.50)	2,720.53 (1,010.43–7,324.91)	10,637.74	11.06 (9.29)	2,129.33 (929.07)
Acute motor axonal neuropathy	10	1,509.67 (775.35–2,939.47)	1,506.76 (774.71–2,930.52)	1,3041.24	10.35 (8.65)	1,305.99 (747.82)
Trigemino-cardiac reflex	7	1,204.39 (549.10–2,641.72)	1,202.76 (548.87–2,635.65)	7,486.01	10.07 (8.36)	1,071.32 (555.26)
Glossoptosis	19	1,079.59 (671.73–1,735.12)	1,075.63 (670.31–1,726.04)	18,380.44	9.92 (8.24)	969.29 (651.67)
Central sleep apnoea syndrome	7	707.73 (328.49–1,524.81)	706.78 (328.36–1,521.28)	4,601.4	9.36 (7.67)	659.27 (346.85)
Floppy iris syndrome	21	623.90 (401.04–970.58)	621.37 (400.09–965.02)	12,230.84	9.19 (7.52)	584.36 (403.73)
Phaeochromocytoma crisis	7	524.05 (244.87–1,121.49)	523.34 (244.78–1,118.88)	3,464.28	8.96 (7.27)	496.84 (262.87)
Postresuscitation encephalopathy	3	498.28 (156.15–1,590.03)	498.00 (156.16–1,588.09)	1,415.99	8.89 (7.18)	473.95 (179.50)
Upper airway obstruction	37	498.61 (357.96–694.52)	495.05 (356.21–688.00)	17,365.35	8.88 (7.21)	471.28 (357.15)
Diabetes insipidus	75	389.00 (308.35–490.74)	383.37 (304.88–482.08)	27,526.81	8.53 (6.86)	368.97 (303.78)
Recurrence of neuromuscular blockade	5	379.98 (155.45–928.79)	379.61 (155.43–927.13)	1,817.61	8.51 (6.83)	365.48 (173.01)
Cardiac arrest neonatal	4	350.05 (129.07–949.37)	349.78 (129.07–947.93)	1,343.17	8.40 (6.71)	337.76 (146.57)
Intestinal pseudo-obstruction	29	347.47 (239.69–503.72)	345.53 (238.83–499.89)	9,623.04	8.38 (6.71)	333.79 (244.64)
Withdrawal hypertension	6	338.11 (149.77–763.32)	337.72 (149.73–761.74)	1,947.21	8.35 (6.67)	326.50 (165.18)
Airway complication of anaesthesia	7	336.52 (158.34–715.20)	336.07 (158.28–713.53)	2,260.91	8.34 (6.67)	324.95 (172.92)
Mechanical ventilation complication	3	299.99 (95.07–946.58)	299.81 (95.08–945.42)	866.91	8.18 (6.49)	290.94 (111.23)
Central venous pressure increased	3	277.35 (88.01–874.02)	277.19 (88.01–872.95)	802.85	8.07 (6.39)	269.58 (103.18)
Mean arterial pressure decreased	5	245.08 (100.86–595.53)	244.85 (100.85–594.46)	1,184.65	7.90 (6.22)	238.90 (113.65)
Neonatal hypotension	8	242.95 (120.40–490.23)	242.57 (120.34–488.95)	1,878.12	7.89 (6.21)	236.74 (131.57)
Sinus arrest	30	233.43 (162.36–335.62)	232.08 (161.75–333.00)	6,743.02	7.82 (6.16)	226.73 (167.33)
Arteriospasm coronary	65	211.78 (165.40–271.18)	209.13 (163.83–266.97)	13,183.35	7.68 (6.01)	204.78 (166.52)
Hypocapnia	6	170.53 (76.05–382.39)	170.33 (76.03–381.60)	992.75	7.39 (5.71)	167.43 (85.19)
Product closure removal difficult	11	164.83 (90.77–299.29)	164.48 (90.69–298.29)	1,757.83	7.34 (5.67)	161.78 (98.21)
Sedation complication	30	162.20 (112.96–232.90)	161.26 (112.54–231.08)	4,700.79	7.31 (5.64)	158.66 (117.22)
Delayed recovery from anaesthesia	12	157.91 (89.21–279.50)	157.54 (89.12–278.49)	1,837.07	7.28 (5.61)	155.06 (96.17)
Hyperthermia malignant	19	156.03 (99.09–245.69)	155.46 (98.89–244.39)	2,870.35	7.26 (5.59)	153.05 (104.67)
Rhythm idioventricular	4	149.64 (55.72–401.84)	149.53 (55.72–401.23)	581.26	7.20 (5.53)	147.29 (64.45)
Drug withdrawal convulsions	22	141.06 (92.52–215.06)	140.46 (92.29–213.77)	3,003.3	7.11 (5.45)	138.49 (97.31)
Epidermolysis bullosa	3	134.24 (42.95–419.59)	134.16 (42.95–419.08)	391.16	7.05 (5.37)	132.36 (51.01)
Atrioventricular dissociation	3	123.01 (39.38–384.23)	122.94 (39.38–383.76)	358.34	6.92 (5.25)	121.42 (46.82)

Due to the potential confounding effect of variations in baseline data on the reliability of disproportionate analysis results (de Vries et al., 2020), sensitivity analyses were undertaken. These analyses encompassed age stratifications (<18 years, 18–65 years, >65 years), gender categorization (male, female), and body weight consideration (subgroups with <50 kg, 50–100 kg, and subgroups >100 kg were omitted due to underreporting) aimed at enhancing result precision.

Withdrawal hypertension (*n* = 4; ROR 808.29; PRR 804.9; IC 9.12; EBGM 557.54) exhibited a significant signal in the <18 years group (Supplementary Figure S1) but was absent from the top 30 adverse event signals in both the 18–65 years group (Supplementary Figure S2) and >65 years group (Supplementary Figure S3). Conversely, in the >65 years group, the most pronounced signal pertained to central sleep apnea syndrome (*n* = 6; ROR 5829.52; PRR 5798.77; IC 11.82; EBGM 3624.6). Moreover, across all age subgroups, bradycardia was the most frequently reported adverse drug reaction among the top 30 signals.

Gender disparities might affect the sensitivity to dexmedetomidine-associated sedation (Vincent et al., 2023). Hence, we conducted subgroup analyses to examine the potential influence of gender on dexmedetomidine-associated adverse effects among men and women. The outcomes are delineated in Supplementary Figures S4, S5. Noteworthy adverse events particular to the male subgroup included transcranial electrical motor evoked potential abnormalities, acute motor axonal neuropathy, central sleep apnea syndrome, cardiac arrest neonatal, postresuscitation encephalopathy, intestinal pseudo-obstruction, hypocapnia, withdrawal hypertension, atrioventricular dissociation, and epidermolysis bullosa.

High-risk adverse drug events specific to the female subgroup comprise pheochromocytoma crises, recurrence of neuromuscular blockade, airway complication of anaesthesia, tachyphylaxis, laryngospasm, cerebral artery occlusions, thyrotoxic crises, drug withdrawal convulsions, bradyarrhythmias, and atrioventricular block second degree.

Finally, we performed similar sensitivity analyses to assess the effect of body weight on adverse drug reactions signal in different subgroups (Supplementary Figures S6, S7). Our results suggested that glossoptosis is the symptom that signals the strongest adverse effect in the 50–100 kg group, with diabetes insipidus following only behind. In contrast, arteriospasm coronary showed significant signal strength in the <50 kg subgroup.

The subgroup analyses described above provide important insights for refining strategies for the clinical use of dexmedetomidine, enabling healthcare professionals to develop appropriate early warning treatment plans for adverse drug events based on the specific characteristics of the corresponding subgroups.

## 4 Discussion

Dexmedetomidine, a highly selective α2-adrenergic agonist, induces sedation and dose-dependent hypnotic-anesthetic action by acting on α2 receptors in the central nucleus of the locus coeruleus, leveraging its unique pharmacological properties to activate endogenous sleep-promoting neural circuits (Doze et al., 1989; Weerink et al., 2017; Belur Nagaraj et al., 2020). This sedation, distinct from other sedatives, preserves a natural non-rapid eye movement sleep state with minimal respiratory impact (Purdon et al., 2015). Additionally, dexmedetomidine possesses anxiolytic and analgesic properties, making it well-suited for intensive care, surgical sedation, and pain management (Anger, 2013). Its mechanism of action, involving the hyperpolarization of noradrenergic neurons leading to reduced norepinephrine release, distinctively modulates pain and stress responses (Yu et al., 2018). In recent years, dexmedetomidine has gradually gained attention for its organ-protective role related to anti-inflammatory responses. Numerous animal experiments have demonstrated that dexmedetomidine reduces the expression of serum and tissue inflammatory mediators (Li et al., 2021; Zhang et al., 2021; Han et al., 2022). Dexmedetomidine can reduce neuroinflammation in neurological disorders by mediating anti-inflammatory effects in microglia (Yamazaki et al., 2022). The mechanisms of action include the upregulation of microglial anti-inflammatory polarization and the reduction of microglial expression of M1-related inflammatory marker genes (Sun et al., 2018; Qiu et al., 2020).

Sedation management, crucial in treating agitation and anxiety in critically ill patients, aims to achieve a state where patients are sedated yet cooperative, easily aroused, and able to communicate their needs, particularly regarding analgesia (Stollings et al., 2022). Dexmedetomidine is effectively used for sedating mechanically ventilated patients in intensive care units (Hughes et al., 2021), providing surgical sedation, and serving as an anesthetic adjunct to enhance analgesia and reduce anesthetic requirements (Mahmoud and Mason, 2015). Additionally, sublingual dexmedetomidine has been approved for treating schizophrenia and acute agitation in bipolar disorder (Citrome et al., 2022; Preskorn et al., 2022). With the increasing clinical use of dexmedetomidine (Liu et al., 2021; Kong et al., 2023), its safety profile remains a focus, and ongoing real-world studies monitoring its adverse effects are essential for ensuring medication safety.

Prior safety studies on dexmedetomidine have often been constrained to single clinical trial data, lacking a comprehensive representation of real-world scenarios due to strict trial designs. In this study, we conducted a systematic evaluation of dexmedetomidine-related adverse reactions using extensive real-world data, analyzing the FAERS database from 2004 to the third quarter of 2023. By employing an ADR signal calculation method, the study not only clarified existing descriptive information about dexmedetomidine but also identified new potential safety risks, thereby providing detailed and reliable data for its future clinical application.

With the expansion of approved indications and the increased use of dexmedetomidine, there has been a notable rise in its adverse reaction reports from 2019 to Q3 2023, comprising 53.72% of total reports, underscoring the need for serious consideration of dexmedetomidine-related adverse reactions. Concurrently, the utilization of dexmedetomidine in sedation during custodial care has been increasingly recognized amidst the backdrop of the COVID-19 pandemic from 2019 to 2023. Previous studies have demonstrated that dexmedetomidine significantly reduces mortality and effectively treats COVID-19-related acute respiratory distress syndrome (ARDS) in patients afflicted with COVID-19 (Hamilton et al., 2021; Zhao et al., 2021; Simioli et al., 2023). Nonetheless, managing COVID-19-related ARDS frequently necessitates prolonged periods of invasive ventilation and heightened sedation levels, potentially resulting in aberrant hemodynamic variability and delirium onset (Bernard-Valnet et al., 2022). Investigating multimodal sedation regimens, such as the combination of ketamine and dexmedetomidine, offers a potential avenue to attain accelerated sedation onset and establish a more consistent hemodynamic state (Riccardi et al., 2023). Our analysis reveals that the majority of these reports (94.9%) were submitted by healthcare professionals, likely due to the prevalence of cardiac disorders as major adverse reactions, necessitating vigilant medical supervision. Additionally, the predominance of reports from the United States (43%) suggests regional variations in adverse reaction profiles, influenced by local expert consensus and other factors. A significant limitation in our study was the absence of specific timing data for a large proportion of adverse reactions (90.79%), restricting our investigation into the time to onset. The following section discusses specific clinical adverse reactions associated with dexmedetomidine:

Our analysis identified a range of adverse reactions associated with dexmedetomidine, affecting a total of 26 organ systems. Consistent with the drug’s insert, the primary focus of dexmedetomidine-associated adverse reactions was the cardiovascular system (Piao and Wu, 2014). And notably, our study found that endocrine system disorders also have high-intensity signals, such as diabetes insipidus. In line with existing literature (Kraus et al., 2023), dexmedetomidine is frequently implicated in sedation-related diabetes insipidus in critically ill ICU patients. Potential mechanisms include dexmedetomidine’s reduction of central arginine vasopressin (AVP) release and diminished renal response to AVP in canine and rat models (Rouch and Kudo, 1996; Kudo et al., 1999; Villela et al., 2005). When ICU patients exhibit diabetes insipidus symptoms, ongoing dexmedetomidine use should be considered in the differential diagnosis. However, given the limited case reports and studies, further large-scale prospective cohort studies are warranted to elucidate its mechanistic effects on diabetes insipidus.

From the data mining process, 892 dexmedetomidine-associated risk signals (Preferred Terms, PTs) were identified. To minimize false positives and enhance detection accuracy, only PTs with three or more reported cases were selected, resulting in 346 PTs included in our analysis. The most frequently reported adverse reactions to dexmedetomidine were bradycardia, cardiac arrest and hypotension (Piao and Wu, 2014; Lewis et al., 2022), consistent with our findings and attributable to its central sympatholytic effects. These adverse effects underscore the importance of vigilant monitoring of patients' hemodynamic parameters and prompt management of complications during dexmedetomidine administration, particularly in patients with cardiac insufficiency. Beyond the anticipated adverse events, our study also uncovered some unexpected adverse events, such as abnormal transcranial electrical stimulation motor evoked potential monitoring, acute motor axonal neuropathy, and trigeminal cardiac reflex, which merit further investigation and evaluation.

Real-time intraoperative monitoring of motor evoked potentials (MEPs) via transcranial electrical stimulation is crucial for assessing the integrity of motor nervous system pathways and reducing the risk of neurological injury (Legatt et al., 2016). The impact of dexmedetomidine on intraoperative neuromonitoring remains a subject of debate. While some studies suggest avoiding dexmedetomidine in children undergoing posterior spinal fusion surgery (PSFS) to prevent interference with neurophysiological monitoring (Mahmoud et al., 2010; Holt et al., 2020; Abdelaal Ahmed Mahmoud Metwally Alkhatip et al., 2023), other research indicates that dexmedetomidine as an anesthetic adjuvant does not significantly affect somatosensory or motor evoked potential responses in complex spinal surgeries (Bala et al., 2008). This study’s findings indicate that abnormal transcranial electrical stimulation motor evoked potential monitoring may be a potential adverse event associated with perioperative dexmedetomidine use, shedding light on its clinical risks.

Acute motor axonal neuropathy (AMAN), a subtype of Guillain-Barre syndrome (GBS), often presents with autonomic dysfunction, including unstable blood pressure and heart rate (Hamel and Logigian, 2023), which can influence anesthesia choices. Additionally, case reports indicate that conditions mimicking AMAN, such as certain neuropathies, may lead to misdiagnosis, complicating anesthetic management (Fodale et al., 2005; Xu et al., 2021). Therefore, it is crucial for clinicians to be aware of the patient’s medical history and to conduct thorough preoperative neurological function assessments.

The trigeminal cardiac reflex (TCR), a prevalent brainstem reflex in maxillofacial neurosurgery, involves the trigeminal nerve, vagus nerve, and central brainstem nuclei, leading to symptoms like hemodynamic changes, apnea, and hypergastricity (Chowdhury et al., 2015; Schaller and Chowdhury, 2021). Dexmedetomidine’s central sympatholytic effect, which suppresses the sympathetic nervous system and reduces sympathetic activity in the heart, can result in TCR, often manifesting as peripheral vasodilation, decreased heart rate, and reduced blood pressure (Bond et al., 2016; Arnold et al., 2018). Minimizing dexmedetomidine use and enhancing intraoperative hemodynamic monitoring are potential strategies for managing TCR during procedures that may trigger it.

The <18 years subgroup analyses indicated that the signal intensity of hemodynamic-related adverse events was more pronounced. Previous studies have shown that during dexmedetomidine infusion, hypotension occurs in 27%–53% of pediatric patients, bradycardia in 21%–25%, and hypertension in 27%–53% (Carney et al., 2013; Banasch et al., 2018). These results suggest that the use of dexmedetomidine in pediatric patients requires careful monitoring of adverse hemodynamic events. Additionally, in the >65 years subgroup, central sleep apnea syndrome warrants clinical attention. A case report suggests that the combined use of perioperative opioids and dexmedetomidine may trigger central sleep apnea syndrome (Ho et al., 2005). Moreover, descriptive baseline population data suggest proportional differences in the gender distribution of adverse effects. Basic studies have demonstrated that gender differences influence the anxiolytic and sedative effects of dexmedetomidine (Jang et al., 2019; Vincent et al., 2023). Identifying biological or social factors associated with gender may provide guidance for monitoring dexmedetomidine adverse reactions.

It is crucial to note that the discussion of dexmedetomidine’s adverse events and their potential mechanisms is based on preliminary analyses of existing literature and data mining. The occurrence and reporting of adverse events are influenced by various factors, including drug properties, individual patient differences, and underlying health conditions. Consequently, establishing exact causality necessitates further large-scale, multicenter clinical studies. Furthermore, drug-induced adverse reactions frequently correlate with dosage, formulation, and administration methods. Research indicates a decreased incidence of adverse cardiovascular events with perioperative dexmedetomidine administration at a push dose below 0.5 μg kg^−1^ or continuous infusion without a push (Demiri et al., 2019). Conversely, higher rates of bradycardia and hypotension were observed in recipients of dexmedetomidine at push doses of 0.75 or 1.0 μg kg^−1^ compared to those receiving 0.5 μg kg^−1^ (Kim et al., 2013). Employing perioperative continuous low-dose infusion and minimizing push administration may mitigate adverse effects. Moreover, findings from a pharmacologic clinical trial revealed a 30% likelihood of specific adverse events with sublingual film administration of dexmedetomidine at doses of 120 μg or 180 μg, despite its efficacy in reducing agitation scores. Given that the FAERS database primarily comprises self-reported adverse events, data gaps exist, such as standardized documentation of dosage and route of administration. Consequently, additional clinically oriented studies are imperative to elucidate the pathogenesis of these adverse reactions. Meanwhile, healthcare professionals are advised to continue vigilant monitoring of adverse events during the clinical use of dexmedetomidine and to implement timely interventions.

While this study offers scientific analyses of real-world data for evaluating the safety of dexmedetomidine from multiple perspectives, there are inherent limitations. First, the reliance on voluntary reporting to the FAERS database may result in incomplete data, lacking of detailed clinical information on patients, such as comorbidities, underlying diseases, and relevant medication history. Second, reporter bias could affect data quality, potentially leading to overrepresentation of certain rare nonclinical adverse events. Third, the analysis of disproportionate data is limited to assessing the strength of the adverse reaction signal and does not allow for quantification of risk or identification of drug-related causation. Finally, to support more prudent use of dexmedetomidine in the future, large-scale prospective studies combining clinical trials with epidemiologic studies are recommended. This study would provide a more reliable evidence-based rationale for the safe use of dexmedetomidine and inform further clinical practice.

## 5 Conclusion

Our analysis of dexmedetomidine’s adverse event reports, sourced from the FAERS database, and our results suggest that dexmedetomidine-associated cardiovascular adverse reactions are common and require focused attention, accounting for 24.59% in addition to the total number of overall adverse reactions. In addition, our study highlighted clinical adverse events with rare but significant signal intensity, including diabetes insipidus and trigeminal cardiac reflexes. This research enriches our understanding of dexmedetomidine’s safety profile, aiding healthcare professionals in making informed treatment decisions. While the FAERS database offers extensive data on drug-related adverse events, its reliance on voluntary reporting and susceptibility to reporting bias necessitates careful interpretation of these findings. Nevertheless, our preliminary results improve the understanding of the drug safety of dexmedetomidine, support effective clinical management in pharmacovigilance studies, and provide important insights for optimizing drug use regimens.

## Data Availability

The original contributions presented in the study are included in the article/Supplementary Material, further inquiries can be directed to the corresponding author.
