# Supplementary material for: Clinical adverse events to dexmedetomidine: a real-world drug safety study based on the FAERS database
Source: Front Pharmacol. 2024 Jul 2;15:1365706. doi: 10.3389/fphar.2024.1365706 (PMC11250259; doi:10.3389/fphar.2024.1365706)
Supplement: Supplementary file 1 [file DataSheet1.PDF]

Supplementary Material

1.1 Supplementary Tables

Supplementary Table 1 Contingency table for adverse events signal detection.

	Dexmedetomidine related ADEs	Non-Dexmedetomidine related ADEs	Total
Dexmedetomidine related ADEs	a	b	a+b
Non-Dexmedetomidine related ADEs	c	d	c+d
Total	a+c	b+d	N=a+b+ c+d

a, number of reports containing both dexmedetomidine and the target related adverse drug events;

b, number of reports containing other adverse drug events of dexmedetomidine;

c, number of reports containing the target related adverse drug events of other drugs;

d, number of reports containing other drugs and other adverse drug events;

Abbreviations: ADEs, adverse drug events.

Supplementary Table 2 ROR, PRR, BCPNN, and EBGM methods, formulas, and thresholds.

Method	Formula	Threshold
ROR	ROR=(ad)/(bc)<br>95%CI = eln(ROR) ± 1.96√(1/a + 1/b + 1/c + 1/d)	the lower limit of 95% CI > 1, a ≥ 3
PRR	PRR= [a(c + d)]/[c(a + b)]<br>95%CI = eln(PRR) ± 1.96√(1/a - 1/(a+b) + 1/c -1/(c+d))	the lower limit of 95% CI > 1, a ≥ 3

BPCNN	$IC = \log 2\ (aN)/(a + b)\ (a + c)$<br>$95\%CI = E(IC) \pm 2 \times \sqrt{V(IC)}$	IC025 > 0 (IC025: the lower bound of 95% CI)
MGPS	$EBGM = (aN)/[(a + b)(a + c)]$<br>$EBGM05 = e^{ln(EGBM) - 1.64\sqrt{(1/a + 1/b + 1/c + 1/d)}}$	EBGM05 > 2 (EBGM05: the lower bound of 95% CI)

Abbreviations: ROR, reporting odds ratio; PRR, proportional reporting ratio; BCPNN, bayesian confidence propagation neural network; MGPS, multi-item gamma Poisson shrinker; EBGM,empirical Bayesian geometric mean; CI, confidence interval; IC, information component.

1.2 Supplementary Figures

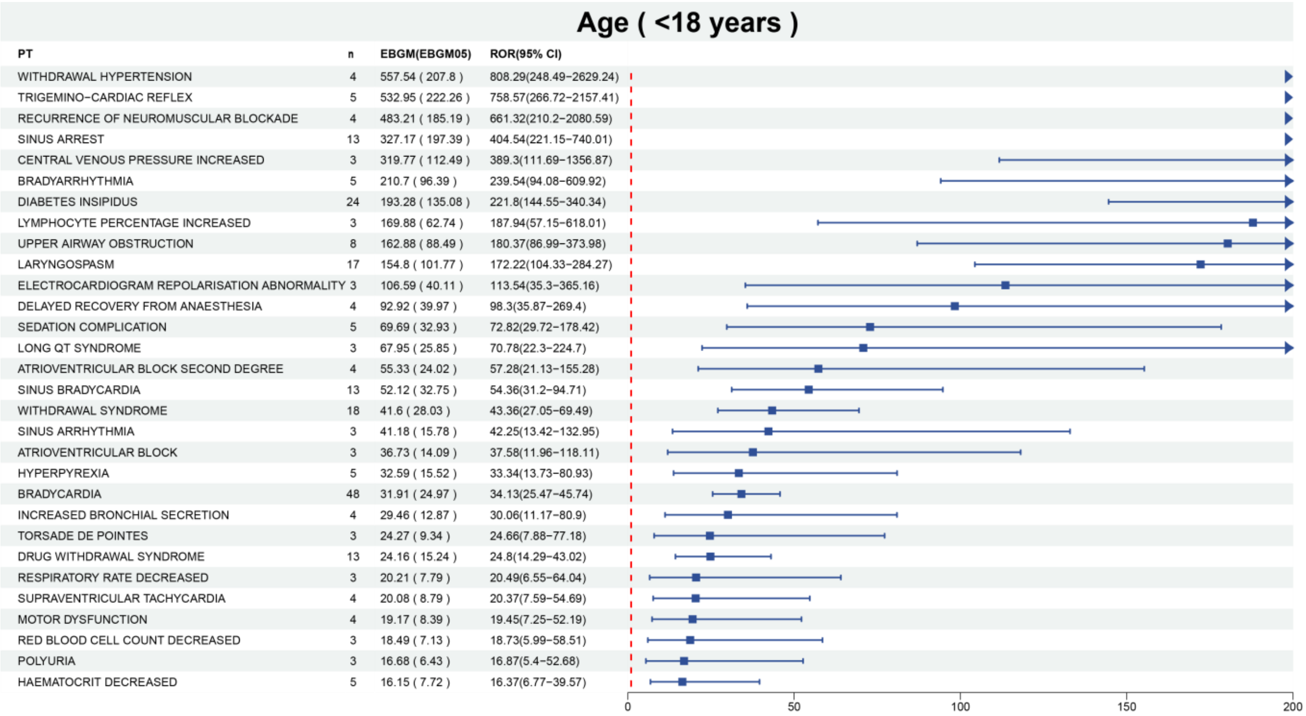

Supplementary Figure 1. The top 30 clinical adverse reactions of Dexmedetomidine ranked by EBGM at the PTs level, stratified by age <18. The red dotted line indicates that the ROR value is equal to 1. The arrows signify that the 95% confidence interval of the ROR exceeds 200.

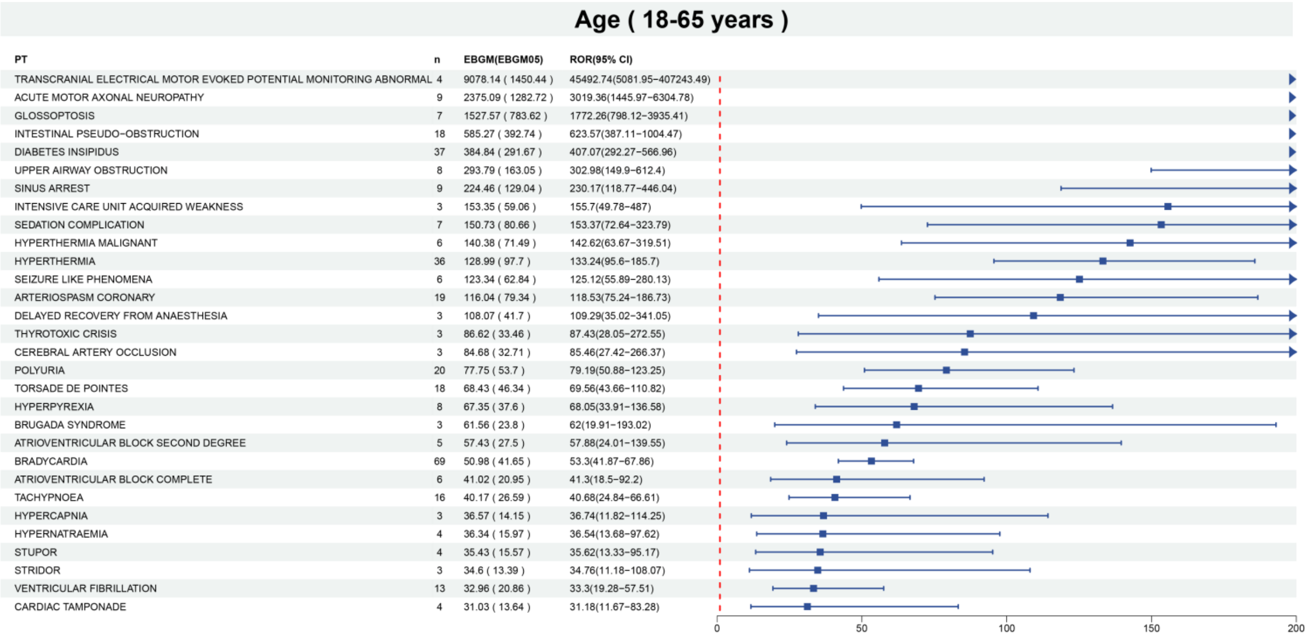

Supplementary Figure 2. The top 30 clinical adverse reactions of Dexmedetomidine ranked by EBGM at the PTs level, stratified by age 18-65. The red dotted line indicates that the ROR value is equal to 1. The arrows signify that the 95% confidence interval of the ROR exceeds 200.

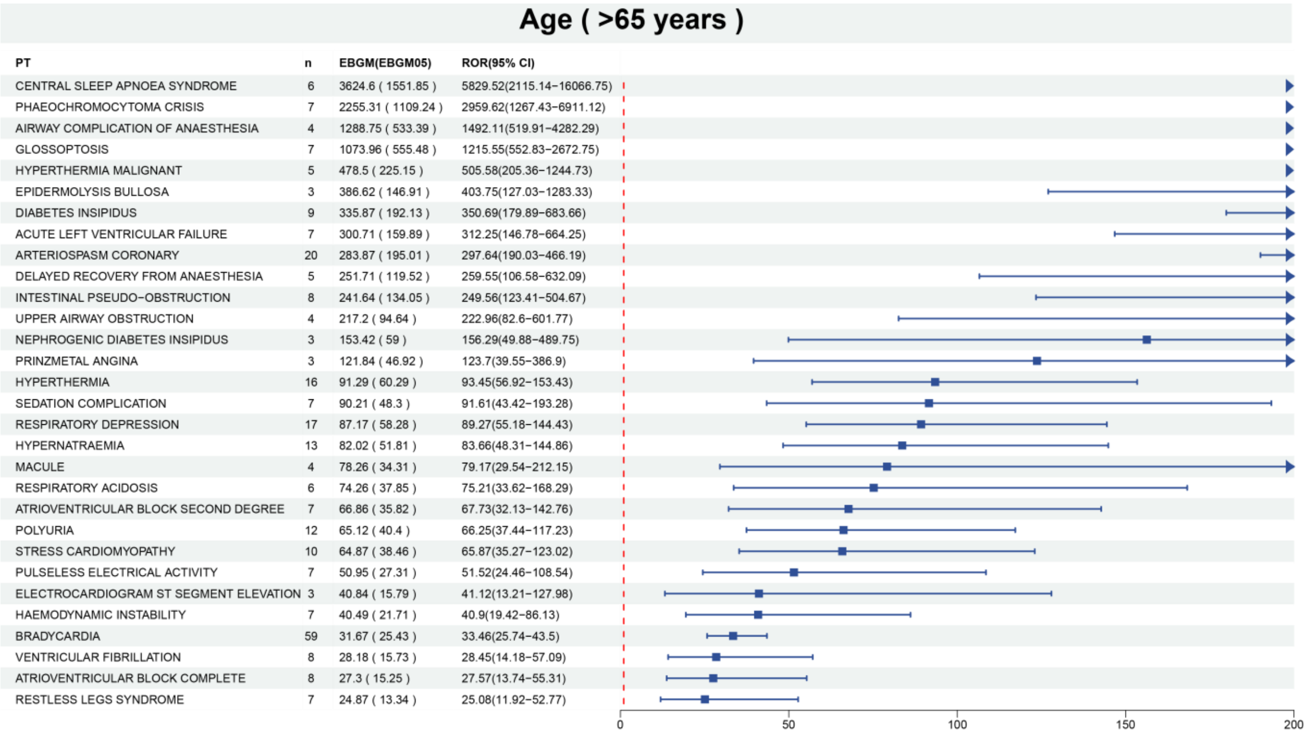

Supplementary Figure 3. The top 30 clinical adverse reactions of Dexmedetomidine ranked by EBGM at the PTs level, stratified by age >65. The red dotted line indicates that the ROR value is equal to 1. The arrows signify that the 95% confidence interval of the ROR exceeds 200.

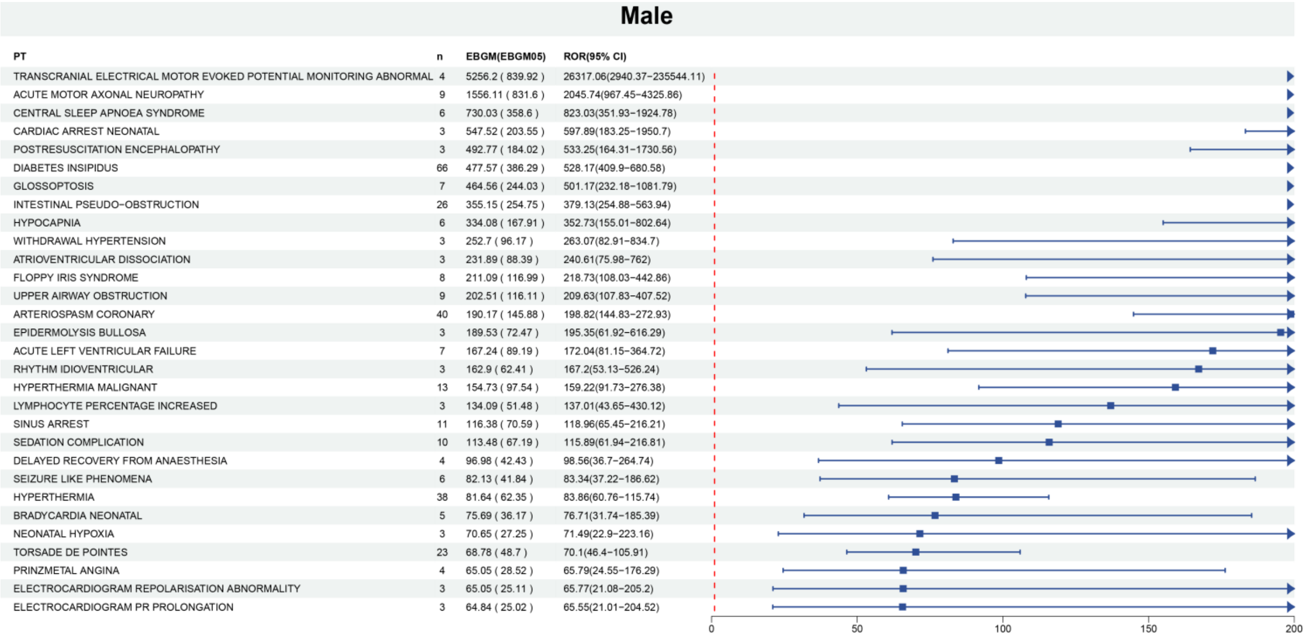

Supplementary Figure 4. The top 30 clinical adverse reactions of Dexmedetomidine ranked by EBGM at the PTs level, stratified by male. The red dotted line indicates that the ROR value is equal to 1. The arrows signify that the 95% confidence interval of the ROR exceeds 200.

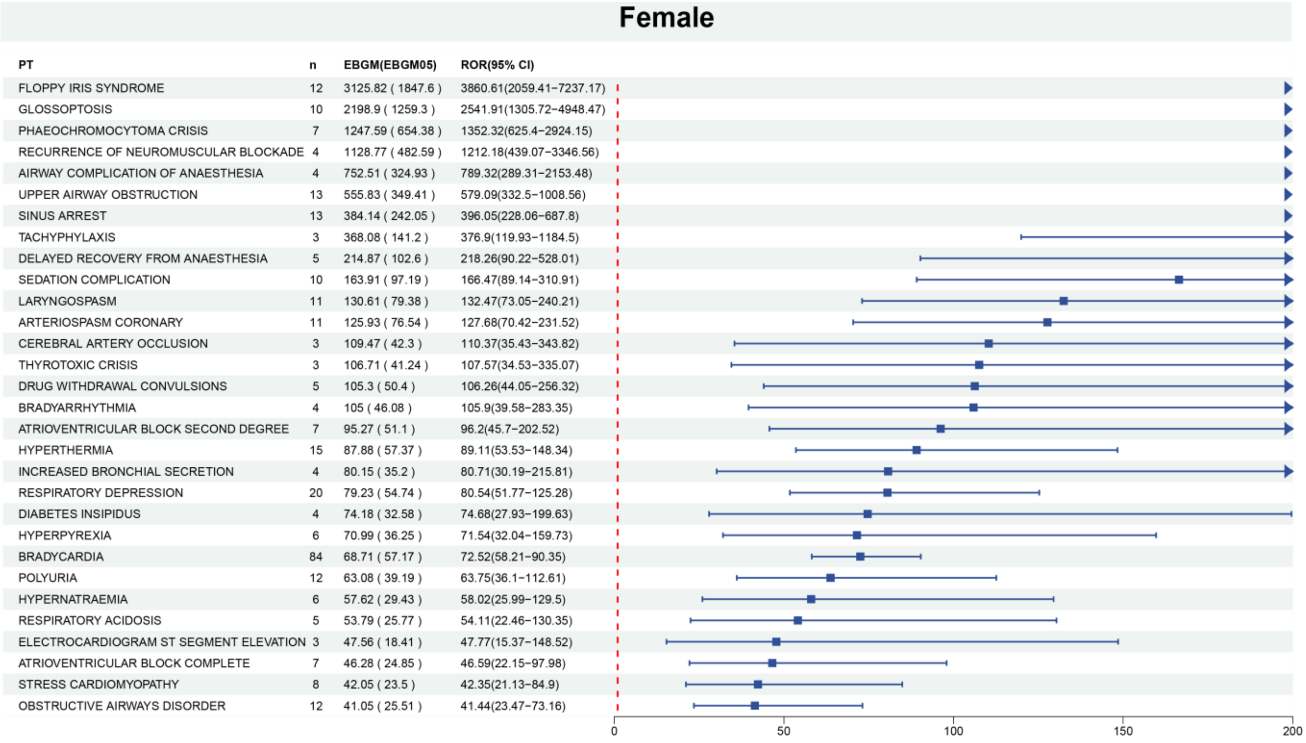

Supplementary Figure 5. The top 30 clinical adverse reactions of Dexmedetomidine ranked by EBGM at the PTs level, stratified by female. The red dotted line indicates that the ROR value is equal to 1. The arrows signify that the 95% confidence interval of the ROR exceeds 200.

Weight (>50kg)

PT	n	EBGM(EBGM05)	ROR(95% CI)
GLOSSOPTOSIS	8	2283.97 (1194.08)	2861.86(1318.36−6212.45)
DIABETES INSIPIDUS	32	1121.47 (822.76)	1284.06(886.8−1859.28)
EPIDERMOLYSIS BULLOSA	3	605.45 (228.64)	640.47(200.01−2050.89)
UPPER AIRWAY OBSTRUCTION	4	298.22 (129.73)	307.3(113.64−831.01)
HYPERTHERMIA MALIGNANT	4	225.1 (98.18)	230.48(85.5−621.29)
SEDATION COMPLICATION	3	215.44 (82.72)	220.16(70.13−691.12)
PRINZMETAL ANGINA	3	162.57 (62.56)	165.38(52.82−517.79)
TORSADE DE POINTES	14	161.77 (103.7)	166.5(97.86−283.28)
ARTERIOSPASM CORONARY	9	148.38 (85.37)	151.72(78.36−293.74)
DELAYED RECOVERY FROM ANAESTHESIA	3	142.75 (54.98)	144.96(46.34−453.42)
ATRIOVENTRICULAR BLOCK SECOND DEGREE	6	102.83 (52.37)	104.39(46.61−233.79)
POLYURIA	14	94.23 (60.49)	96.42(56.76−163.79)
MACULE	3	87.14 (33.63)	88.06(28.23−274.7)
HYPERNATRAEMIA	6	81.86 (41.72)	82.95(37.07−185.64)
HYPERTHERMIA	9	69.17 (39.87)	70.24(36.36−135.69)
RESPIRATORY DEPRESSION	8	65.95 (36.78)	66.88(33.28−134.39)
BRADYCARDIA	46	51.72 (40.34)	54.58(40.56−73.46)
HYPOXIC−ISCHAEMIC ENCEPHALOPATHY	3	48.57 (18.78)	48.92(15.71−152.32)
ATRIOVENTRICULAR BLOCK COMPLETE	5	42.47 (20.33)	42.85(17.76−103.35)
PROTHROMBIN TIME PROLONGED	6	40.06 (20.44)	40.45(18.1−90.4)
CARDIAC TAMPONADE	3	39.06 (15.11)	39.31(12.63−122.34)
HAEMOPHAGOCYTIC LYMPHOHISTIOCYTOSIS	3	36.58 (14.15)	36.81(11.83−114.53)
PULSELESS ELECTRICAL ACTIVITY	3	35.58 (13.76)	35.8(11.5−111.38)
CARDIAC ARREST	34	31.71 (23.8)	32.96(23.39−46.44)
VENTRICULAR FIBRILLATION	6	29.9 (15.26)	30.16(13.5−67.37)
NEUROLEPTIC MALIGNANT SYNDROME	4	29.82 (13.1)	30.02(11.23−80.26)
BLOOD ALBUMIN DECREASED	5	28.83 (13.81)	29.05(12.05−70.03)
ATRIOVENTRICULAR BLOCK	3	24.9 (9.64)	25.03(8.05−77.85)
OBSTRUCTIVE AIRWAYS DISORDER	4	24.46 (10.75)	24.61(9.21−65.78)
ACIDOSIS	3	23.97 (9.28)	24.09(7.75−74.92)

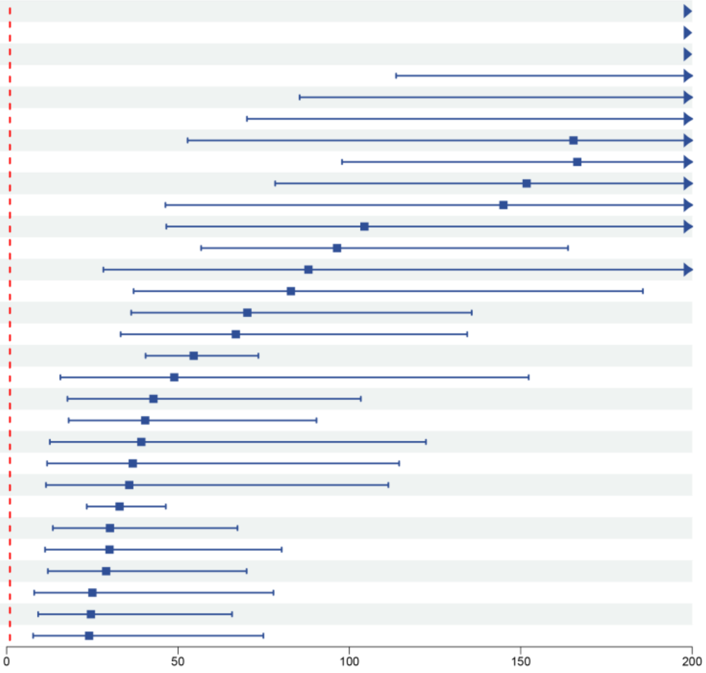

Supplementary Figure 6. The top 30 clinical adverse reactions of Dexmedetomidine ranked by EBGM at the PTs level, stratified by weight >50 kg. The red dotted line indicates that the ROR value is equal to 1. The arrows signify that the 95% confidence interval of the ROR exceeds 200.

Weight (<50kg)

PT	n	EBGM(EBGM05)	ROR(95% CI)
ARTERIOSPASM CORONARY	10	420.07 (235.08)	527.54(263.6−1055.74)
GLOSSOPTOSIS	5	330.97 (148.91)	392.75(151.2−1020.17)
UPPER AIRWAY OBSTRUCTION	6	262.13 (128.04)	300.36(127.57−707.16)
THYROTOXIC CRISIS	3	242.71 (88.73)	274.12(82.35−912.47)
CARDIAC ARREST NEONATAL	4	189.95 (80.34)	209.16(74.79−584.9)
SEDATION COMPLICATION	6	179.54 (89.01)	197.25(85.27−456.26)
SINUS ARREST	4	164.86 (70.1)	179.28(64.52−498.15)
NODAL RHYTHM	3	133.74 (50.23)	143.02(44.37−460.95)
DELAYED RECOVERY FROM ANAESTHESIA	3	78.01 (29.68)	81.22(25.59−257.74)
PULSELESS ELECTRICAL ACTIVITY	4	61.53 (26.72)	63.65(23.49−172.5)
NEONATAL RESPIRATORY DEPRESSION	3	52.85 (20.22)	54.37(17.25−171.36)
BRADYPNOEA	3	49.27 (18.87)	50.6(16.07−159.34)
BRADYCARDIA	46	48.52 (37.67)	53.1(39.24−71.84)
RESPIRATORY DEPRESSION	10	43.95 (25.93)	45.48(24.21−85.46)
RESPIRATORY ACIDOSIS	3	31.21 (12)	31.78(10.14−99.59)
SINUS BRADYCARDIA	4	28.74 (12.56)	29.28(10.89−78.74)
OBSTRUCTIVE AIRWAYS DISORDER	4	26.8 (11.72)	27.28(10.15−73.33)
SEDATION	6	26.64 (13.54)	27.18(12.11−61.02)
HYPERTHERMIA	4	25.04 (10.95)	25.46(9.47−68.41)
BRADYCARDIA NEONATAL	7	24 (12.83)	24.5(11.59−51.8)
MIOSIS	3	23.74 (9.14)	24.09(7.7−75.36)
POLYURIA	3	22.44 (8.65)	22.76(7.28−71.16)
DRUG WITHDRAWAL SYNDROME	8	21.82 (12.14)	22.27(11.06−44.88)
WITHDRAWAL SYNDROME	8	21.42 (11.92)	21.86(10.85−44.04)
POSTERIOR REVERSIBLE ENCEPHALOPATHY SYNDROME	4	17.13 (7.5)	17.36(6.47−46.55)
NEONATAL HYPOXIA	3	16.89 (6.51)	17.08(5.47−53.34)
CARDIAC ARREST	17	16.39 (10.94)	16.9(10.42−27.4)
CIRCULATORY COLLAPSE	4	14.78 (6.48)	14.96(5.58−40.11)
HYPOALBUMINAEMIA	4	13.44 (5.89)	13.59(5.07−36.43)
SINUS TACHYCARDIA	3	12.58 (4.86)	12.7(4.07−39.59)

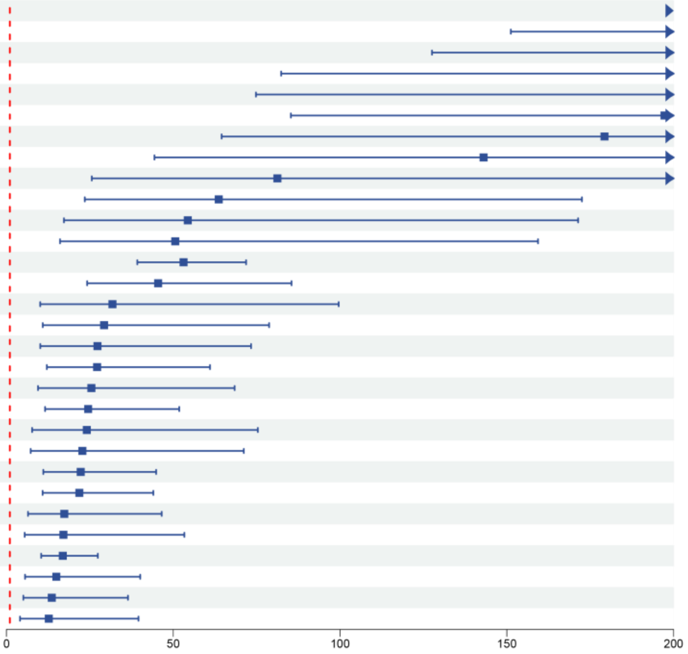

Supplementary Figure 7. The top 30 clinical adverse reactions of Dexmedetomidine ranked by EBGM at the PTs level, stratified by weight <50 kg. The red dotted line indicates that the ROR value is equal to 1. The arrows signify that the 95% confidence interval of the ROR exceeds 200.
